# Berberine Damages the Cell Surface of Methicillin-Resistant *Staphylococcus aureus*

**DOI:** 10.3389/fmicb.2020.00621

**Published:** 2020-04-28

**Authors:** Xiujuan Zhang, Xiaoying Sun, Jiaxin Wu, Yue Wu, Yali Wang, Xiaoqing Hu, Xiaoyuan Wang

**Affiliations:** ^1^State Key Laboratory of Food Science and Technology, Jiangnan University, Wuxi, China; ^2^Key Laboratory of Quality and Standard Research of Traditional Chinese Medicine in Gansu Province, Gansu University of Traditional Chinese Medicine, Lanzhou, China; ^3^School of Food Science and Engineering, Wuhan Polytechnic University, Wuhan, China; ^4^International Joint Laboratory on Food Safety, Jiangnan University, Wuxi, China

**Keywords:** berberine, cell surface, *Staphylococcus aureus*, damage, integrity, fatty acids

## Abstract

Methicillin-resistant *Staphylococcus aureus* (MRSA) is currently regarded as one of the most important drug-resistant pathogens causing nosocomial and community-acquired infections. Although berberine (BER) has shown anti-MRSA activity, the underlying mechanism is still unclear. In this study, the damage caused by BER on the cell surface of MRSA was systematically investigated by performing BER susceptibility test, determining K^+^ and alkaline phosphatase (ALP) release, detecting morphological alterations using scanning electron microscopy (SEM) and transmission electron microscopy (TEM), and ascertaining lipid profiles. The results showed that the minimum inhibitory concentration (MIC) of BER against MRSA252 was 128 μg/ml. Under the sub-MIC doses of BER, cell membrane permeability gradually increased in a dose-dependent manner, and 1 × MIC led to 43.8% higher K^+^ leakage and fourfold higher ALP secretion. The injuries on MRSA cell surface were further verified by SEM and TEM, and some cells displayed a doughnut-shaped structure. BER significantly altered the fatty acid species contents, including saturated fatty acids (C_14__:__0_, C_15__:__0_, C_16__:__0_, C_18__:__0_, and C_20__:__0_), and unsaturated fatty acids (C_20__:__4_, C_20__:__1_, and C_18__:__1_), indicating that BER compromised cell membrane integrity via lipid fluctuation. Thus, the findings of this study could help to unravel the molecular mechanism of BER against MRSA.

## Introduction

The recent emergence and worldwide spread of multidrug-resistant bacteria have raised major public health concerns. The data released by the Center for Disease Control and Prevention indicate that at least 2 million illnesses and 23,000 deaths are caused by multidrug-resistant bacteria every year in the United States. For example, methicillin-resistant *Staphylococcus aureus* (MRSA), a main cause of nosocomial and community-acquired infections around the world (Hoffman and Outterson, 2015), have developed multidrug resistance and, thus, have been responsible for substantial morbidity and mortality in hospitals (Yu et al., 2005). Nowadays, single-drug treatments are becoming less efficient for curing MRSA infections (Luo et al., 2014), and therefore, MRSA has been classified as one of the most serious pathogenic threats around the world.

Methicillin-resistant *Staphylococcus aureus* was identified as one of the global priority pathogens with antibiotic resistance by the World Health Organization (WHO) in 2017 (Tacconelli et al., 2018), and thus, had attracted increasing concerns worldwide. Therefore, there is an urgent need to develop anti-MRSA agents to treat invasive and life-threatening infections (Yang et al., 2019a, b). Accordingly, some natural antibiotic substances have been explored as candidates to control MRSA (Yu et al., 2005). Among these antibacterial agents, berberine (BER), one of the major components of berberine alkaloids from *Coptis chinensis* Franch, has been noted to show broad-spectrum antimicrobial activity against a variety of microorganisms (Wu et al., 2011), including MRSA (Luo et al., 2014). BER has been reported to display antimicrobial activity against almost all the tested MRSA strains, with minimum inhibitory concentrations (MIC) ranging from 32 to 128 μg/ml (Yu et al., 2005).

However, although BER (Yu et al., 2005; Luo et al., 2013; Chu et al., 2016) or its derivatives (Wang et al., 2017; Yang et al., 2019a, b) had been confirmed to possess anti-MRSA activities, knowledge about the underlying mechanism is still limited. Moreover, previous studies only focused on the biofilm state, but not on the planktonic state of MRSA. Yu et al. (2005) reported that 1–50 μg/ml of BER inhibited MRSA adhesion to human gingival fibroblasts, which is the first and crucial step during biofilm development. The effect of BER on MRSA adhesion was dose dependent. Chu et al. (2016) indicated that BER inhibited MRSA biofilm formation by affecting the aggregation of phenol-soluble modulins, which are small peptides and the main components of the extracellular amyloid fibril required for MRSA biofilm formation. By molecular dynamics simulation, it was revealed that BER might bind to the phenyl ring of Phe19 in PSMα2 through hydrophobic interactions (Chu et al., 2016).

In another work on the antifungal activity of BER from medically important plants of barberry species against *Candida* sp., Zoric et al. (2017) reported that BER affected the synthesis of membrane ergosterol and increased membrane permeability. In addition, BER was noted to cause membrane depolarization and lipid peroxidation, which was supported by the increase in ROS levels. These results suggested that BER could damage the cell surface. However, there is still a lack of detailed understanding of the effect of BER on the cell surface, such as the morphological changes and fatty acid profiles.

To obtain more evidence of cell surface injuries in MRSA and elucidate the mechanism of BER against MRSA, transmission electron microscopy (TEM) and scanning electron microscopy (SEM) were employed to reveal the morphological alteration in MRSA following BER treatment. Furthermore, fatty acid profiles were analyzed to understand changes in lipid profiles following BER treatment. To the best of our knowledge, this is the first report on MRSA cell surface damage caused by BER treatment. Besides, these findings could help to better understand the detailed bactericidal mechanisms of BER against MRSA.

## Materials and Methods

### Bacterial Strain and Growth Conditions

MRSA252 was incubated on Luria–Bertani (LB) agar plates (containing 10 g/L of tryptone, 5 g/L of yeast extract, 10 g/L of NaCl, 15 g/L of agar, and pH of 7.0) at 37°C for 1–2 days. Then, a single MRSA colony was picked and transferred into 10 ml of LB medium (without agar) in a shake flask and cultivated for 5–10 h at 37°C and 200 rpm, until an optical density at 600 nm (OD_600_) of 1 (Wu et al., 2018).

### Anti-MRSA Test

The MIC of BER was defined as the lowest concentration of BER causing inhibition of MRSA growth in 24 h and was determined by a 24-h growth inhibitory assay (Wojtyczka et al., 2014), with minor modifications as follows. First, BER was dissolved in sterile water at 80°C and serially diluted (5,120, 2,560, 1,280, 640, 320, 160, 80, 40, and 20 μg/ml). Then, the growth inhibitory assays were conducted in sterile test tubes containing 6 ml of reaction mixture, consisting of 400 μl of logarithmic-phase MRSA cell suspension at a final density of 5 × 10^6^ colony-forming unit (CFU)/ml and 600 μl of BER solution (final concentrations of 2, 4, 8, 16, 32, 64, 128, 256, and 512 μg/ml, respectively). After cultivation at 37°C for 24 h, the cells were harvested by centrifugation at 5,000 × *g* and 4°C for 10 min and washed with 0.1 M phosphate buffer solution (PBS) at pH 7.4, and the dried biomass was analyzed. The test was conducted five times, and the results were presented as the mean ± SD (*N* = 5). In subsequent investigations, the sub-MIC doses of BER were employed in most cases to investigate the cell surface damage.

### Detection of K^+^ and Alkaline Phosphatase Leakages

To assess the extent of intracellular content release that reflects the permeability of the outer membrane of MRSA, the K^+^ concentration and alkaline phosphatase (ALP) activity were determined respectively. The extracellular K^+^ leakages in the BER-treated and control cells were assayed after 9 h, as described previously (Wu et al., 2018), and the ALP activity in the culture broth was evaluated using an ALP kit (Nanjing Jiancheng Institute of Bioengineering, Nanjing, Jiangsu, China) and a microplate reader (Eon, Biotek, United States) (Cao et al., 2019; Kang et al., 2020).

### SEM and TEM Observation

After incubation, the cells were harvested by centrifugation for 10 min at 5,000 × *g* and 4°C, and rinsed three times with 0.1 M PBS at pH 7.4. The pellet was collected, and the cells were fixed with 2.5% glutaraldehyde in 0.1 M PBS at 4°C for 12 h. Then, the cells were harvested by centrifugation at 5,000 × *g* and 4°C for 5 min, and subjected to gradual dehydration with ethanol (30, 50, 70, 80, 90, and 100%) for 10 min, respectively. Finally, the specimens were sputter-coated with gold under vacuum and subjected to microscopic examinations using Tescon Mira3 SEM, as described previously (Lv et al., 2011). For TEM observations, the MRSA252 cells treated with BER were harvested by centrifugation at 5,000 × *g* and 4°C for 10 min and processed as reported previously (Shi et al., 2017). The ultrathin sections were examined under a JEOL 2100F microscope (Hitachi, Tokyo, Japan).

### Quantification of ROS and Lipid Peroxidation

The cells were incubated in sterile test tubes containing 6 ml of reaction mixture, consisting of 400 μl of logarithmic-phase MRSA cell suspension (final cell density of 5 × 10^6^ CFU/ml) and 600 μl of BER solution (final concentration of 1 × MIC). ROS and lipid peroxidation were quantified according to the previously described method (Wu et al., 2018).

### Analysis of Fatty Acids From the Sub-Lethal Bacteria

Fatty acids were extracted from BER-treated and control MRSA according to the previously described method (Wu et al., 2018), with some minor modifications as follows: after cooling, the pH was adjusted to two with HCl, and 3 ml of anhydrous diethyl ether was added.

## Results

### Bactericidal Activity of BER

**FIGURE 1:**
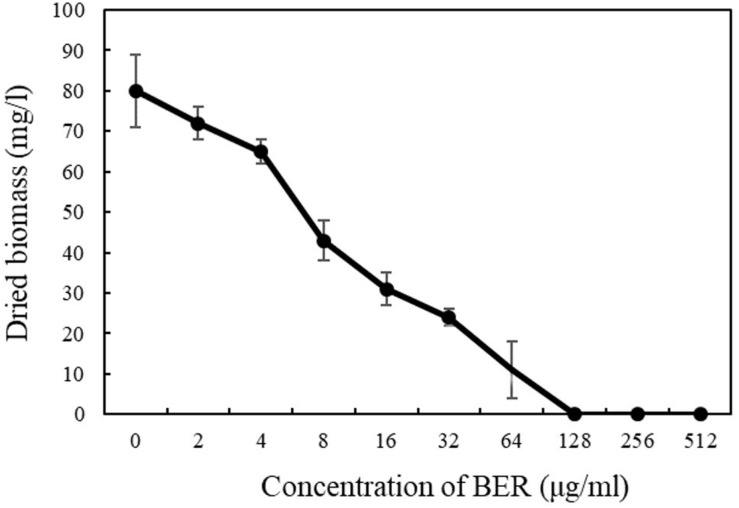
Biomass of MRSA252 after 24 h cultivation exposed to BER with different concentrations. Data are means, and bars represent standard deviations.

The antimicrobial efficacy of BER against MRSA was evaluated by biomass changes after BER treatment. As shown in Figure 1, the susceptibility test showed that the MIC of BER against MRSA252 was 128 μg/ml, similar to those indicated in previous reports (Chu et al., 2016). The MRSA252 growth was completely inhibited at BER concentrations ≥128 μg/mL, and the subsequent investigations were mostly conducted at sub-MIC doses.

### Modulation of Cell Membrane Permeability in BER-Treated MRSA Cells

**FIGURE 2:**
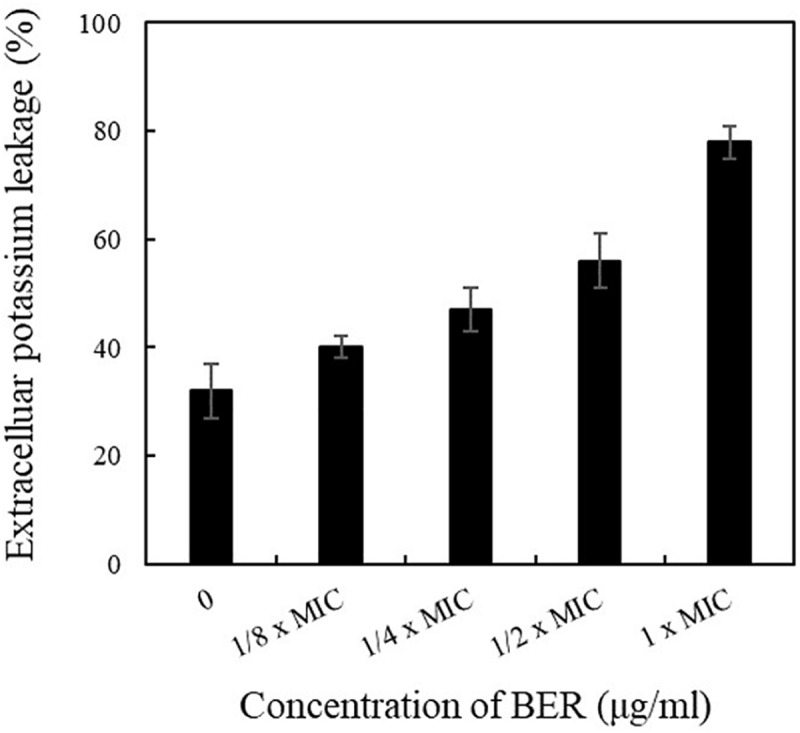
Effects of different doses of BER on the leakage rate of K^+^ of MRSA252. Data are means, and bars represent standard deviations.

Following the determination of MIC value, the influences of BER on the permeability and integrity of MRSA cell membranes were further investigated. The extracellular K^+^ content was analyzed using flame atomic absorption spectrometry at 766.5 nm. The results showed that intracellular components were released in BER-treated cells in a dose-dependent manner. As shown in Figure 2, the control cells without BER treatment exhibited normal K^+^ leakage at about 30%, while all the BER-treated cells presented higher K^+^ leakage. Higher BER doses led to more significant K^+^ leakage, and 1 × MIC resulted in 78% K^+^ leakage, which was 43.8% higher than that noted in the control, indicating serious injuries of the plasma membrane. Thus, more severe permeabilization of the plasma membrane might be achieved with 2× and 4 × MIC of BER, leading to disruption of cells during the initial growth phase. Similar trends had also been reported by Zoric et al. (2017).

**FIGURE 3:**
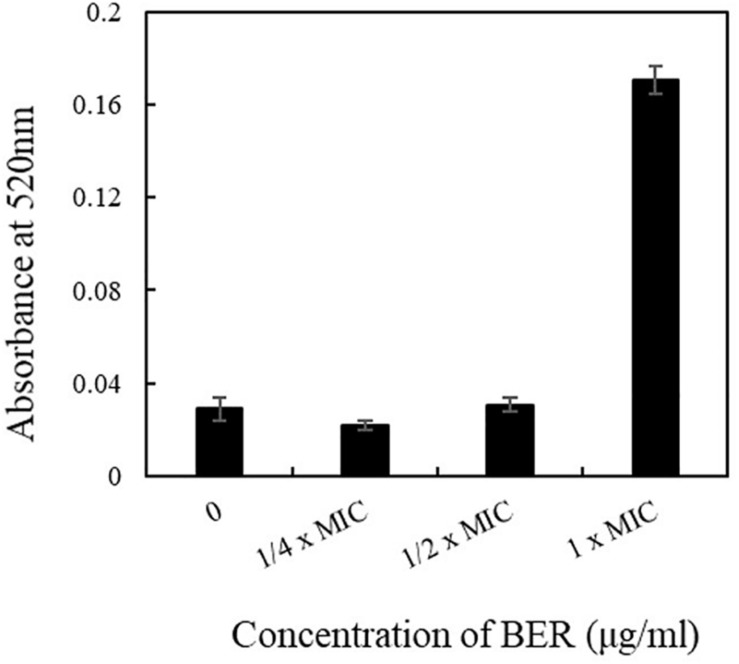
Effects of different doses of BER on the activities of alkaline phosphatases released to the broth. Data are means, and bars represent standard deviations.

In addition, periplasmic ALP was also employed as an indicator of membrane permeability changes. As ALP would be secreted into the extracellular environment in cases of increased cell surface injuries, its activity could reflect an increase in cell membrane permeability and impaired biogenesis of membrane components. As illustrated in Figure 3, the ALP activity after 12 h remained stable when BER dose ranged from 1/4 × MIC to 1/2 × MIC, whereas it sharply increased to fourfold higher level when 1 × MIC BER was employed, indicating an obvious enhancement in membrane permeability and release of cellular contents. In particular, BER increased the permeability of cell membrane and deteriorated the integrity of the cell surface, as indicated by few supportive evidences. The SEM and TEM images revealed the morphological changes in MRSA cells following BER treatment.

### Morphological Changes in BER-Treated MRSA Cells

**FIGURE 4:**
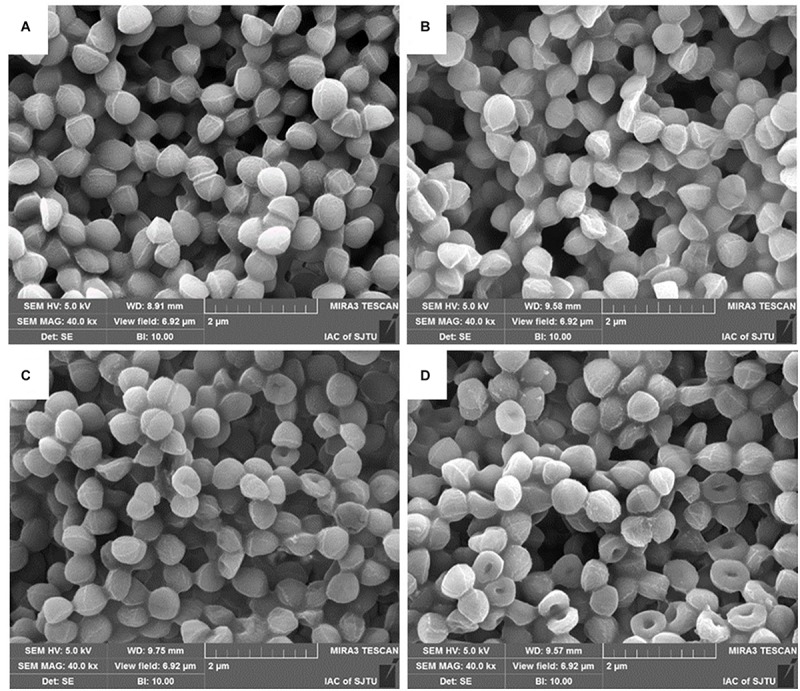
SEM images of MRSA cells treated by BER at 0 × MIC **(A)**, 1/4 × MIC **(B)**, 1/2 × MIC **(C)**, and 1 × MIC **(D)**.

To further evaluate whether the integrity of the MRSA cell wall had been disrupted by BER, we first observed BER-treated cells under SEM to visualize the effects of BER on whole cells. Changes in the morphology of MRSA upon treatment with different doses of BER are displayed in Figure 4. When compared with the control, the BER-treated MRSA cells became non-spherical, with some exhibiting a doughnut-shaped structure without a central hole. With an increase in BER doses, more cells displayed the doughnut-like shape and were significantly different from the control, indicating that BER induced more severe changes in the cell envelope.

**FIGURE 5:**
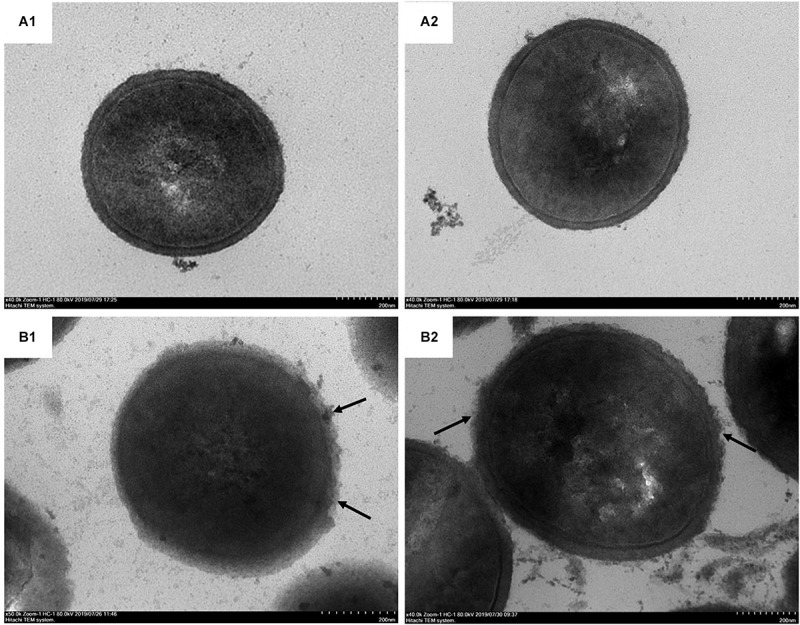
TEM photographs of MRSA cells treated by BER at 0 × MIC **(A1, A2)** and 1 × MIC **(B1, B2)**. BER damaged the intact cell surface (arrow).

In addition to SEM, TEM was also used to directly observe the morphological and ultrastructural changes in MRSA cells after BER treatment. As shown in Figure 5, the control cells were surrounded by a very thick cell wall (Figures 5A1,A2), implying an intact cell envelop, whereas MRSA cells exposed to 1/2 × MIC BER displayed obscure boundaries of the cell wall (Figures 5B1,B2). These pronounced morphological changes suggested the initiation of cell surface injuries by BER, which was also confirmed by the significant release in cellular contents around the cells.

Such direct evidence suggested that the anti-MRSA effect of BER starts with the disruption of the cell wall and increased the permeability of the cell membrane, followed by the disturbance of cellular integrity and the release of intracellular components.

### Fatty Acid Profiles of BER-Treated MRSA Cells

In *Candida* sp., ROS levels have been found to increase following BER treatment, suggesting that ROS could be the most likely factor responsible for cell membrane permeabilization (Zoric et al., 2017). Therefore, in the present study, the levels of intracellular ROS and its oxidative product malondialdehyde were monitored. The results (data not shown) showed an increase in ROS and malondialdehyde levels in BER-treated cells similar to that noted in *Candida* sp. (Zoric et al., 2017), thus confirming the role of ROS in cell membrane damage caused by BER.

Subsequently, fatty acids were extracted from BER-treated and control MRSA cells, respectively, and analyzed by GC-MS to compare the different fatty acid distribution patterns. As shown in Table 1, the overall profiles of fatty acids were distinctly different. When compared with the control cells, the BER-treated cells had significantly lower contents of different fatty acid species, including saturated fatty acids (C_14__:__0_, C_15__:__0_, C_16__:__0_, C_18__:__0_, and C_20__:__0_) and unsaturated fatty acids (C_20__:__4_, C_20__:__1_, and C_18__:__1_). It is known that unsaturated fatty acids are more susceptible to oxidation, when compared with saturated fatty acids; thus, as expected, the content of C_20__:__4_, C_20__:__1_, and C_18__:__1_ decreased following BER treatment. However, the content of saturated fatty acids also decreased because ROS had non-specific targets. Lipid peroxidation (data not shown) changed the fundamental lipid composition of the cell membrane, which affected the physiological characteristics and integrity of cell membranes, such as fluidity, uptake, secretion, and permeability (Hagve, 1988). Taken together, BER-induced MRSA inactivation was mediated by lipid fluctuations and subsequent cell surface damage. To the best of our knowledge, this is the first report demonstrating that BER caused a reduction in unsaturated fatty acid contents. The results of the present study established that treatment of MRSA cells with BER compromised bacterial cell wall integrity via lipid fluctuations, thus, leading to cell disruption and suggesting a potential role of BER in combating MRSA infection.

**TABLE 1:** The change of fatty acid profiles in MRSA252 upon BER treatment.

**Retention time (min)**	**Cellular fatty acids**	**Percentage (%)**	
		**0 × MIC**	**1 × MIC**
7.3922	C_14:0_	0.54 ± 0.02	0.06 ± 0.03
8.3612	C_15:0_	13.89 ± 1.22	1.11 ± 0.05
9.3034	C_16:0_	8.68 ± 0.29	0.11 ± 0.04
11.1027	C_18:0_	5.93 ± 0.32	0.15 ± 0.03
11.2004	C_18:1_	0.20 ± 0.02	0.09 ± 0.02
12.8763	C_20:1_	0.05 ± 0.01	ND
13.0762	C_20:4_	0.65 ± 0.09	0.30 ± 0.04
13.5697	C_20:0_	0.17 ± 0.22	0.55 ± 0.03

*ND, not detectable.*

## Discussion

Nowadays, drug resistance among pathogens is recognized as one of the most important global public health problems, which threatens the treatment of many infectious diseases. The emergence of various “superbugs” that possess increasing antibiotic resistance represents a growing health and economic burden. Among these drug-resistant bacteria, MRSA is regarded as one of the most important pathogens causing nosocomial and community-acquired infections. The risk of mortality and medical costs (Song et al., 2017) of MRSA infections are very high because single-drug treatments of nosocomial and community-acquired infections are becoming less efficient (Song et al., 2017). In addition, the presence of MRSA biofilms in the food industry poses a serious risk to food contamination (Yan et al., 2017). Therefore, along with the steady rise in antibiotic-resistant bacteria, more and more researchers are seeking alternative therapies instead of conventional medicine, such as Chinese herbal therapy.

*Rhizoma coptidis* has been used in traditional Chinese medicine to combat diarrhea, fever, and jaundice for more than 2,000 years, and has shown significant anti-MRSA activity (Luo et al., 2014). Recently, a variety of effective components were isolated from *R. coptidis*, and their susceptibility to MRSA was tested (Yu et al., 2005; Luo et al., 2014). Berberine alkaloids, such as BER, coptisine, palmatine, epiberberine, and jatrorrhizine, have been proven to be the major effective components inhibiting MRSA. BER is a bright yellow isoquinoline alkaloid, rich in the root, rhizome, and stem bark of medically important plants of Barberry species. Furthermore, BER has been traditionally used for many years in India and China as antimicrobial medicine, and has been demonstrated to be a strong synergist for antibiotic treatments (Chu et al., 2016).

Several researchers had explored the action mechanism of BER against *S. aureus* (Inna et al., 2001; Wang et al., 2008). Inna et al. found that the plant cations BER can penetrate the phospholipid bilayers of the bacterial membrane. Even if a part of them was extruded by a multidrug resistance pump, BER can still be accumulated by *S. aureus* cell membrane (Inna et al., 2001), while its influences on cell membrane stability needed further investigation. In addition, genome-wide transcription profiles of *S. aureus* cells before and after BER treatment were compared by Wang et al. (2008) to reveal the global change in bacteria, and BER evidently elevated transcripts of five genes such as urease-encoding genes and *kdpABC* genes involved in transport of potassium cations, and depressed that of six genes. Especially, the transcriptions of several surface protein-encoding genes *isdCDEFGI* involved in hemoglobin binding and passage of heme-iron to the cytoplasm were downregulated obviously. The Isd (iron-regulated surface determinant) system was critical for the essential nutrient of *S. aureus* (Skaar and Schneewind, 2004), and IsdC, IsdDEF, and IsdGI encoded the cell wall-anchored heme binding protein, membrane transport system, and two cytoplasmic heme-degrading monooxygenases, respectively (Wang et al., 2008). Although BER could have possibly altered the amount of some membrane proteins and deteriorated the cell membrane, it is still unclear whether these changes led to damage in cell membrane integrity and disruption of bacterial cell. In the current study, BER exerted an anti-MRSA effect at a dose ≥128 μg/ml (MIC value). The efficacy of BER against MRSA252 incited our interest to further investigate its underlying mechanism against MRSA. The most obvious morphological characteristic of BER-treated MRSA cells was the dose-dependent change in the cell membrane. This observation is consistent with the previous findings indicating that BER inhibited the growth of MRSA, which was confirmed by a damage in the cell surface by TEM and SEM. These findings revealed that BER exerted anti-MRSA activity by damaging the cell surface and releasing the intracellular contents.

As a non-traditional approach to combat drug-resistant bacteria, targeting cell membrane and destroying cell membrane adaptation are considered as novel antimicrobial strategies (Tran et al., 2016). The drastic oxidation reaction on the cell membrane and/or cell wall could obviously produce negative impacts on bacterial viability, resulting in lipid peroxidation, protein and polysaccharide oxidation, alterations to cell permeability, etc. (Tran et al., 2016). The integrity of the cell membrane is crucial for bacterial survival. The ROS such as H_2_O_2_ and OH^∗^ would exert noxious effects such as peroxidation of polyunsaturated fatty acids, which was one of the causes for cell membrane injury (Tirlapur et al., 2001). The oxidation of polyunsaturated lipids was initiated by hydrogen atom abstraction at bis-allylic sites and induced a chain reaction. As a consequence, various toxic products were generated and damaged the stability of the cell membrane (Andreyev et al., 2015). In the present study, BER was shown to affect fatty acid profiles and cell permeability in MRSA cells. Xue et al. (2015) investigated the antibacterial activity of BER from *Coptidis rhizoma* on the cell membrane of *Aeromonas hydrophila* and revealed that the permeability of the cell membrane was increased by 19.66%, which was elevated evidently, while the underlying mechanism was unknown. Our experiments revealed for the first time that the alteration of fatty acids may be the primary cause of the increase in cell membrane permeability. Additionally, Zoric et al. (2017) found that ROS was generated following BER addition, and BER obviously upregulated MDA levels, which might be one of the *Candida-cidal* mechanisms. The correlation between BER treatment and ROS increase was also found in the present study (data not shown), and this may be the most possible reason for changes in fatty acids profiles. Based on the results of BER susceptibility assays, cell membrane permeability, ultrastructural observations, and fatty acid profiles, it was hypothesized that unsaturated fatty acids might be the target of BER-induced ROS, which deteriorated the cell integrity. These findings provide an insight into the molecular mechanism of BER for anti-MRSA therapies. Based on the current work and previous publications, ROS generated after BER treatment may be the direct inducer of lipid fluctuation. How BER induced ROS is an interesting question and will be investigated systematically in our future work.

## Data Availability Statement

The raw data supporting the conclusions of this manuscript will be made available by the authors, without undue reservation, to any qualified researcher.

## Author Contributions

XZ and XS conducted most of the experiments and wrote the manuscript. JW assayed the fatty acids. YWu analyzed the ROS. YWa and XH designed the experiments and modified the manuscript. XW helped to reveal the underlying mechanism.

## Conflict of Interest

The authors declare that the research was conducted in the absence of any commercial or financial relationships that could be construed as a potential conflict of interest.
